# Parenting practices and oral health status in rural areas in Egypt: a household survey

**DOI:** 10.1186/s12903-020-01123-5

**Published:** 2020-05-06

**Authors:** Nourhan M. Aly, Ahmed Abdelrahman Mohamed, Wafaa E. Abdelaziz

**Affiliations:** Department of Pediatric Dentistry and Dental Public Health, Faculty of Dentistry, Alexandria University, Champolion St., Azarita, Alexandria, 21527 Egypt

**Keywords:** Parenting, Oral health, Rural population, Children, Dental caries, Gingivitis, Egypt

## Abstract

**Background:**

Parenting practices influence children’s health and development. The present study assessed the association between parenting practices and oral health status of children living in rural areas in Egypt.

**Methods:**

A cross-sectional household survey including 190 households and 392 children was conducted from May 2019 to January 2020 in four villages in Egypt. Data were collected through clinical examination and interview-based questionnaires of children. Clinical examination assessed caries (DMFT and dft), oral hygiene and gingival condition. Parenting practices were assessed using the short version of the Alabama Parenting Questionnaire (APQ) and oral health practices were assessed using the WHO questionnaire-child form. Four linear regression models were used to assess the relationship between four outcome variables (oral health indicators: (DMF, df, plaque and gingival indices) and parenting practices and oral health behaviors (exposure) after adjusting for potential confounders. Regression coefficients (B), 95% confidence intervals (CI) and model adjusted R^2^ were calculated.

**Results:**

Complete questionnaires and clinical data were available for 392 children (response rate = 86.34%). The mean (SD) age = 9.93 (3.05) with 54.60% females. Most children (67.60%) had caries in their primary teeth, mean ± SD of df = 2.94 ± 3.10, while only 27.30% had caries in their permanent teeth, mean DMF ± SD = 0.57 ± 1.13. There was a statistically significant difference between parenting practices of both fathers and mothers (*p* < 0.001, < 0.001, < 0.001, 0.008 and < 0.001 for the five parenting constructs). The adjusted R^2^ of the models that included parenting practices (for DMF = 0.168, for df = 0.400, for plaque index = 0.061 and for gingival index = 0.090) were similar to the models that included oral health behaviors (for DMF = 0.197, for df = 0.421, for plaque index = 0.059 and for gingival index = 0.084).

**Conclusion:**

The association between oral health status and parenting practices which- although not statistically significant- was similar in impact to that between oral health behaviors and oral health status, highlighting the importance of parenting practices to oral health.

## Background

Childhood is a critical phase in which physical, mental, emotional and social health are established [1]. Children’s health practices are shaped during this period [2]. Parents, intentionally or unintentionally, have a great impact on their child’s health and development. Proper parenting practices lead to positive childhood outcomes and better quality of life because they are significantly associated with children’s diet, sleep and physical exercise [1, 3]. Additionally, greater parental support is associated with better health and health services use [4], while the frequency of parental interaction is directly related to early child development [5].

Previous studies have assessed the association between parenting practices and children’s oral health [1, 6–13] and reported that parents affect children’s oral health practices and sugar intake which may affect their oral health status [1, 14]. Those studies were conducted in schools or pediatric dental centers with findings applicable only to children who attended schools or came for treatment in dental clinics [15]. The practices of one parent, usually the mother, were assessed mostly through self-reporting that potentially introduced biases because of social desirability and under-reporting of negative practices [16]. Only one study assessed parenting practices by asking adolescents, rather than parents, and reported no relationship between parenting practices and dental plaque levels [6]. Some studies reported differences in parenting styles of both parents, which highlights the importance of assessing both parents’ practices [17, 18]. Most previous studies on parenting were conducted in Western or Southeast Asian countries except one study in Saudi Arabia [13]. Culture-specific social norms in some countries emphasize parents’ authority in their children’s lives and thus their influence on children’s developing personalities [19]. Previous parenting and oral health studies were conducted in urban settings. Rural environment, however, differs from urban communities in having higher poverty rates that may add stress on parents and affect their parenting practices. Moreover, extensive social relations make parents more likely to offer and receive advice from others living in the same community rather than from health professionals. In addition, less access to preventive care may make individuals more prone to diseases [20, 21].

In 2014, the Egyptian Ministry of Health and the World Health Organization (WHO) conducted an oral health survey in Egypt that revealed that nearly 70% of children had untreated caries [22]. Children below 15 years of age represented 43.72% of the total Egyptian population in 2019, with 61.17% living in rural areas likely to suffer higher poverty rates than those in urban areas (66% compared to 34%) [23, 24]. Egyptian children living in rural areas may be at increased risk of oral diseases than the general population. It is important to identify and address factors associated with children’s health, including parenting practices.

This study assessed the association between parenting and disciplining practices on one side and oral health status of rural children, on the other side. The study findings would help create oral health risk profile for children living in rural areas to design tailored health education campaigns addressing their problems. The null hypothesis was that parenting practices and disciplining techniques are not associated with oral health status of children.

## Methods

The current study was based on a cross-sectional, household survey assessing the oral health and practices of children and mothers in rural Egypt. Recruitment of participants and data collection were conducted from May 2019 to January 2020. Prior to the study, ethical approval was obtained from the Research Ethics Committee at the Faculty of Dentistry, Alexandria University, Egypt (IRB 00010556 – IORG 0008839). Parental consent and children’s assent to participate in the study were also secured.

The study setting was Northwestern Delta, Egypt including three governorates (Alexandria, Marsa Matruh and Beheira). Alexandria is mostly urban, Marsa Matruh is sparsely populated and Beheira has the greatest size of rural population among the three governorates [25]. Each governorate is divided into administrative centers and these, in turn, are divided into local administrative units, that include either villages or cities. Multistage random sampling was used. In the first stage, the most populated administrative center of North Western Delta was selected to reduce transportation costs since the study was unfunded. This administrative center includes 28 administrative units with a total rural population of 710,672 individuals [26]. In the second stage, the villages in the administrative units were identified from Egypt’s Administrative Units guide [25] and four of them were randomly selected (the number was based on sample size considerations as explained below). In the third stage, a local guide in each village helped in the random selection of eligible households. At the last stage, a cluster sample was used to include all children in the selected households.

Participants were eligible if they were residents of the selected villages, between 6 and 18 years of age, and living with mothers/ female caregivers in the same household. Intellectually disabled and preschool children were excluded because they would not understand the questionnaires. Physically disabled and medically compromised children were excluded because their medical condition may affect their oral health status.

Sample size was planned on 95% confidence level to detect levels of caries similar to what a previous study [27] reported among Egyptian children 3–18 years old (mean deft = 4.21, standard deviation (SD) = 3.21, mean DMFT = 1.04, SD = 1.56). The required number of participants was calculated to range from 366 to 370 children [28]. The number of persons per household in villages of average economic level was reported to be 3.57 [24] so it was assumed that the average family would include a mother and 1.57 children. To ensure representativeness and variation of households, at least 35 households were randomly selected per village (n ≈ 90 children) with four villages eventually selected.

Data were collected through clinical examination and interview-based questionnaire of children. Calibration on caries examination was done for two examiners; inter- and intra-examiner reliability were calculated, and Kappa ranged from 0.84–0.94 indicating excellent agreement between examiners and across time [29]. Caries was assessed using the WHO criteria [30] under daylighting conditions, without magnification or drying, and the total DMFT (permanent dentition) and dft (primary dentition) scores for each participant were calculated. The gingival condition was assessed using the gingival index of Löe and Silness [31] whereas, the oral hygiene condition was assessed using the plaque index of Silness and Löe [32]. Both indices use the same 6 index teeth (upper right 1st permanent molar, upper right lateral incisor, upper left 1st premolar or 1st primary molar, lower left 1st permanent molar, lower left lateral incisor and lower right 1st premolar or 1st primary molar) [31, 32]. Disposable mirrors and ball ended WHO probes #550B were used for clinical examination.

Parenting practices were assessed using the short form of the Alabama Parenting Questionnaire (APQ) [33]. The APQ measures five parenting constructs; parental involvement, supervision and monitoring, use of disciplining techniques, consistency of disciplining, and use of corporal punishment. The child form can be used with children aged 6–18 [33, 34]. Scott et al. [35], developed and validated the short version measuring each parenting construct using three questions. This short form was translated into Arabic and validated in 2015 [36] and was used in the current study. All items were scored on a 5-point scale ranging from 1 (never) to 5 (always). The total score of each construct was the sum of scores of the three questions. Higher scores on the positive parenting and parental involvement domains indicate good parenting practices, whereas higher scores on poor monitoring/supervision, corporal punishment and inconsistent discipline indicate ineffective parenting. Each child was interviewed separately, without his/her parent or caregiver. The child responded to the questionnaire once to assess maternal practices and another time to assess paternal practices. Oral health practices were assessed using the Arabic version [37] of the WHO questionnaire – child form [30]. This questionnaire collected information about the child’s age, parental education and oral health behaviors such as toothbrushing (at least once daily or less), sugar consumption (at least once daily or less) and dental visits during the previous year (at least once or less). The questionnaires were pilot-tested on ten children attending the Pediatric Dentistry Clinic at the Faculty of Dentistry, Alexandria, Egypt, after obtaining parental approval to ensure their appropriateness and estimate the time needed for responding. The questionnaires were then field-tested on seven children who were interviewed in their homes in one of the villages. Their data were not included in the final analysis. Data were collected using an online platform (KoboToolbox) that allows offline data collection with subsequent synchronization when internet access becomes available [38]. All individuals in the household were screened for oral diseases and referred for treatment as needed. A toothbrush and toothpaste were given for each family member and oral hygiene instructions were provided.

### Statistical analysis

The exposure variables were parenting practices and oral health behaviors (toothbrushing, sugar consumption, and dental visits), while the outcome variables were oral health status indicators (caries in primary and permanent teeth, gingival condition and oral hygiene). Potential confounders that were adjusted for in all models included age, gender and mother’s education as an indicator of socioeconomic status in addition to village as place of residence.

Parenting practices of mothers and fathers of the same child were compared using paired t test or Wilcoxon signed rank test depending on normality. Four linear regression models were used with adjustment for confounders. Model 1 included oral health behaviors (toothbrushing, sugar consumption and dental visits). Model 2 included all constructs of mothers’ parenting practices and Model 3 included fathers’ parenting practices. In Model 4, both mothers’ and fathers’ parenting practices were included. Regression coefficients (B), 95% confidence intervals (CI) and model adjusted R^2^ were calculated. Significance was set at *P* < 0.05. Data were analyzed using IBM SPSS for Windows version 25 [39].

## Results

In the four villages, 220 households with 454 children were invited and 190 households with 392 children were included, with average number of participants/village = 98 (86.34% response rate). Table 1 shows that females represented 54.60% of the included children and the mean ± SD age in years = 9.93 ± 3.05. Most mothers were non-educated (44.60%) or completed primary or middle school (43.60%). Most children brushed their teeth less than once daily (83.40%), consumed sugar at least once daily (76.50%) and visited the dentist at least once during the previous year (55.60%). Only 27.30% had caries experience in their permanent teeth, mean ± SD DMFT = 0.57 ± 1.13 whereas 67.60% had caries experience in their primary teeth, mean ± SD dft = 2.94 ± 3.10. The mean ± SD plaque and gingival indices = 1.45 ± 0.55 and 1.16 ± 0.37, respectively.

**Table 1:** Description of demographic profile, oral health behaviors and oral health status of children participating in the study (*n* = 392)

Age (mean ± SD)		9.93 ± 3.05
Gender n (%)	Males	178 (45.40%)
	Females	214(54.60%)
Mother education	Non-educated	175 (44.60%)
	Completed primary and middle school	171 (43.60%)
	Completed high school and higher education	46 (11.60%)
Toothbrushing	Less than once daily	327 (83.40%)
	At least once daily	65 (16.60%)
Dental visits last year	At least once	218 (55.60%)
	Less than once or never	174 (44.4%)
Sugar consumption	Less than once daily	92 (23.50%)
	At least once daily	300 (76.50%)
Permanent caries experience (DMFT)	n (%)	107 (27.30%)
	Mean ± SD	0.57 ± 1.13
	Median (IQR)	0.00 (0.00, 1.00)
Primary caries experience (dft)	n (%)	265 (67.60%)
	Mean ± SD	2.94 ± 3.10
	Median (IQR)	2.00 (0.00, 5.00)
Plaque Index (mean ± SD)		1.45 ± 0.55
Gingival Index (mean ± SD)		1.16 ± 0.37

Table 2 shows that there were statistically significant differences between mothers and fathers in all parenting domains except parental knowledge of the friends their child spends time with (*P* = 0.48). Mothers had significantly better parental involvement and positive parenting (*P* < 0.001), while fathers showed significantly more consistent disciplining, monitoring and less corporal punishment (*P* = < 0.001, 0.008, and < 0.001, respectively).

**Table 2:** Parenting practices of mothers and fathers

Parenting Domain	Item	Mother	Father	*P* Value
		Mean ± SD		
Parental Involvement^a^	You play games or do other fun things with your parent	3.21 ± 1.61	2.83 ± 1.64	< 0.001*
	Your parent asks you about your day in school	4.20 ± 1.41	3.31 ± 1.78	< 0.001*
	Your parent helps you with your homework	3.09 ± 1.85	2.43 ± 1.73	< 0.001*
	Total	10.50 ± 3.30	8.57 ± 3.91	< 0.001*
Positive parenting^a^	Your parent tells you that you are doing a good job	4.43 ± 1.21	3.69 ± 1.74	< 0.001*
	Your parent compliments you when you have done something well	4.69 ± 0.94	4.15 ± 1.52	< 0.001*
	Your parent praises you for behaving well	4.64 ± 1.00	4.28 ± 1.41	< 0.001*
	Total	13.75 ± 2.40	12.12 ± 3.90	< 0.001*
Inconsistent discipline^a^	Your parent lets you out of a punishment early	3.48 ± 1.64	2.74 ± 1.75	< 0.001*
	Your parent threatens to punish you and then do not do it	3.32 ± 1.69	2.35 ± 1.64	< 0.001*
	You talk your parent out of punishing you after you have done something wrong	3.59 ± 1.74	2.98 ± 1.87	< 0.001*
	Total	10.39 ± 3.37	8.07 ± 3.72	< 0.001*
Poor supervision and monitoring^b^	You fail to leave a note or let your parent know where you are going	1.86 ± 1.49	1.67 ± 1.31	< 0.001*
	Your parent let you stay out in the evening past the time you are supposed to be home	1.31 ± 0.93	1.26 ± 0.83	0.02*
	Your parent does not know the friends you are with	1.50 ± 1.23	1.54 ± 1.22	0.48
	Total	4.68 ± 2.76	4.47 ± 2.33	0.008*
Corporal punishment^b^	Your parent spanks you with their hand when you have done something wrong	2.55 ± 1.75	2.12 ± 1.67	< 0.001*
	Your parent hits you with a belt, switch, or other object when you have done something wrong	3.09 ± 1.81	2.61 ± 1.80	< 0.001*
	Your parent slaps you when you have done something wrong	2.88 ± 1.78	2.40 ± 1.78	< 0.001*
	Total	8.53 ± 4.05	7.14 ± 4.23	< 0.001*

^a^Paired t-test was used. ^b^Wilcoxon signed rank test was used

*statistically significant at *p* < 0.05

Higher scores of positive parenting and involvement signify good parenting practices, while higher scores of poor monitoring/supervision, corporal punishment and inconsistent discipline indicate ineffective parenting

In Tables 3, 4, 5 and 6, Model 1 shows the association between the four oral health indicators and oral health behaviors. There was a statistically significant association between toothbrushing and dental visits with mean DMFT (B = − 0.28, 95% CI = (− 0.56, 0.009) and B = − 0.31, 95% CI = (− 0.52, − 0.11), respectively), and between sugar consumption and dental visits with mean dft (B = − 0.58, 95% CI = − 1.16, 0.004 and B = − 0.64, 95% CI = − 1.13, − 0.16, respectively). No association was observed between oral health behaviors and plaque index, while only toothbrushing was significantly associated with gingival index (B = 0.11 and 95% CI = 0.10, 0.21). There was no significant association between parenting practices and the four oral health indicators, except for mothers’ and fathers’ poor monitoring that were significantly associated with less gingivitis in models 2 and 3 of Table 6 (B = − 0.02, 95% CI = − 0.03, − 0.004 and B = − 0.02, 95% CI = − 0.04, − 0.002, respectively and fathers’ positive parenting that was significantly associated with more gingivitis in model 3 of Table 6 (B = 0.01 and 95% CI = 0.001, 0.02).

**Table 3:** Association between parenting practices and DMFT scores of permanent caries experience

Factor		Model 1	Model 2	Model 3	Model 4
		B (95% CI)	B (95% CI)	B (95% CI)	B (95% CI)
Mother’s parenting practices	Involvement	–	0.01 (− 0.02, 0.05)	–	0.001 (− 0.04, 0.04)
	Positive parenting	–	− 0.04 (− 0.09, 0.007)	–	− 0.04 (− 0.09, 0.02)
	Inconsistent discipline	–	0.007 (− 0.03, 0.04)	–	0.01 (− 0.03, 0.05)
	Poor monitoring	–	0.02 (− 0.02, 0.06)	–	0.02 (− 0.05, 0.09)
	Corporal punishment	–	− 0.006 (− 0.03, 0.02)	–	0.001 (− 0.03, 0.04)
Father’s parenting practices	Involvement	–	–	0.03 (− 0.004, 0.06)	0.03 (− 0.009, 0.07)
	Positive parenting	–	–	− 0.02 (− 0.05, 0.02)	− 0.008 (− 0.05, 0.03)
	Inconsistent discipline	–	–	− 0.005 (− 0.04, 0.03)	− 0.008 (− 0.05, 0.03)
	Poor monitoring	–	–	0.03 (− 0.02, 0.08)	0.003 (− 0.09, 0.09)
	Corporal punishment	–	–	− 0.009 (− 0.04, 0.02)	− 0.01 (− 0.05, 0.03)
Toothbrushing frequency	Less than once daily	−0.28 (− 0.56, − 0.009)	–	–	–
	Once or more daily	reference	–	–	–
Dental visits per year	At least once	−0.31 (− 0.52, − 0.11)	–	–	–
	Less than once or never	reference	–	–	–
Sugar consumption	Less than once daily	0.09 (−0.16, 0.34)	–	–	–
	At least once daily	reference	–	–	–
Adjusted R^2^		0.197	0.173	0.174	0.168

Model 1: Oral health practices (dental visits, sugar consumption and toothbrushing) adjusted for village, age, gender and mother education

Model 2: Mothers’ parenting practices adjusted for village, age, gender and mother education

Model 3: Fathers’ parenting practices adjusted for village, age, gender and mother education

Model 4: Both mothers’ and fathers’ parenting practices adjusted for village, age, gender and mother education

**Table 4:** Association between parenting practices and dft scores of primary caries experience

Factor		Model 1	Model 2	Model 3	Model 4
		B (95% CI)	B (95% CI)	B (95% CI)	B (95% CI)
Mother’s parenting practices	Involvement	–	0.007 (− 0.07, 0.09)	–	0.03 (− 0.06, 0.12)
	Positive parenting	–	− 0.06 (− 0.17, 0.05)	–	− 0.05 (− 0.18, 0.08)
	Inconsistent discipline	–	− 0.04 (− 0.12, 0.04)	–	− 0.007 (− 0.10, 0.09)
	Poor monitoring	–	− 0.01 (− 0.10, 0.08)	–	− 0.06 (− 0.24, 0.11)
	Corporal punishment	–	0.04 (− 0.03, 0.10)	–	0.05 (− 0.04, 0.13)
Father’s parenting practices	Involvement	–	–	− 0.03 (− 0.11, 0.05)	−0.05 (− 0.13, 0.04)
	Positive parenting	–	–	−0.007 (− 0.09, 0.07)	0.01 (− 0.08, 0.10)
	Inconsistent discipline	–	–	− 0.05 (− 0.12, 0.03)	−0.05 (− 0.13, 0.04)
	Poor monitoring	–	–	0.003 (−0.11, 0.11)	0.06 (− 0.15, 0.27)
	Corporal punishment	–	–	0.02 (−0.05, 0.09)	−0.01 (− 0.10, 0.07)
Toothbrushing frequency	Less than once daily	−0.05 (− 1.11, 0.17)	–	–	–
	Once or more daily	reference	–	–	–
Dental visits per year	At least once	−0.64 (− 1.13, − 0.16)	–	–	–
	Less than once or never	reference	–	–	–
Sugar consumption	Less than once daily	−0.58 (− 1.16, − 0.004)	–	–	–
	At least once daily	reference	–	–	–
Adjusted R^2^		0.421	0.403	0.404	0.400

Model 1: Oral health practices (dental visits, sugar consumption and toothbrushing) adjusted for village, age, gender and mother education

Model 2: Mothers’ parenting practices adjusted for village, age, gender and mother education

Model 3: Fathers’ parenting practices adjusted for village, age, gender and mother education

Model 4: Both mothers’ and fathers’ parenting practices adjusted for village, age, gender and mother education

**Table 5:** Association between parenting practices and plaque index

Factor		Model 1	Model 2	Model 3	Model 4
		B (95% CI)	B (95% CI)	B (95% CI)	B (95% CI)
Mother’s parenting practices	Involvement	–	0.01 (− 0.005, 0.03)	–	0.01 (− 0.009, 0.03)
	Positive parenting	–	− 0.01 (− 0.04, 0.01)	–	− 0.03 (− 0.05, 0.003)
	Inconsistent discipline	–	− 0.007 (− 0.02, 0.01)	–	− 0.02 (− 0.04, 0.006)
	Poor monitoring	–	− 0.008 (− 0.03, 0.01)	–	0.005 (− 0.03, 0.04)
	Corporal punishment	–	− 0.005 (− 0.02, 0.009)	–	− 0.008 (− 0.03, 0.01)
Father’s parenting practices	Involvement	–	–	0.007 (− 0.01, 0.02)	0.002 (− 0.02, 0.02)
	Positive parenting	–	–	0.007 (− 0.01, 0.02)	0.01 (− 0.007, 0.03)
	Inconsistent discipline	–	–	0.002 (− 0.02, 0.02)	0.01 (− 0.009, 0.03)
	Poor monitoring	–	–	− 0.004 (− 0.03, 0.02)	− 0.02 (− 0.06, 0.03)
	Corporal punishment	–	–	− 0.0003 (− 0.02, 0.02)	0.005 (− 0.01, 0.02)
Brushing frequency	Less than once daily	0.05 (− 0.10, 0.20)	–	–	–
	Once or more daily	reference	–	–	–
Dental visits per year	At least once	0.006 (− 0.10, 0.12)	–	–	–
	Less than once or never	reference	–	–	–
Sugar consumption	Less than once daily	−0.10 (− 0.23, 0.03)	–	–	–
	At least once daily	reference	–	–	–
Adjusted R^2^		0.059	0.058	0.055	0.061

Model 1: Oral health practices (dental visits, sugar consumption and toothbrushing) adjusted for village, age, gender and mother education

Model 2: Mothers’ parenting practices adjusted for village, age, gender and mother education

Model 3: Fathers’ parenting practices adjusted for village, age, gender and mother education

Model 4: Both mothers’ and fathers’ parenting practices adjusted for village, age, gender and mother education

**Table 6:** Association between parenting practices and gingival index

Factor		Model 1	Model 2	Model 3	Model 4
		B (95% CI)	B (95% CI)	B (95% CI)	B (95% CI)
Mother’s parenting practices	Involvement	–	− 0.006 (− 0.02, 0.006)	–	−0.005 (− 0.02,0.009)
	Positive parenting	–	0.009 (−0.008, 0.03)	–	0.002 (− 0.02, 0.02)
	Inconsistent discipline	–	0.0004 (−0.01, 0.01)	–	−0.002 (− 0.02, 0.01)
	Poor monitoring	–	−0.02 (− 0.03, − 0.004)	–	−0.009 (− 0.04, 0.02)
	Corporal punishment	–	−0.004 (− 0.014,0.006)	–	− 0.009 (− 0.02,0.003)
Father’s parenting practices	Involvement	–	–	− 0.004 (− 0.02,0.007)	−0.003 (− 0.02, 0.01)
	Positive parenting	–	–	0.01 (0.001, 0.02)	0.009 (−0.004,0.02)
	Inconsistent discipline	–	–	0.001 (−0.01, 0.01)	0.003 (−0.01, 0.02)
	Poor monitoring	–	–	−0.02 (− 0.04, − 0.002)	−0.01 (− 0.04, 0.02)
	Corporal punishment	–	–	0.003 (−0.007,0.01)	0.01 (− 0.003,0.02)
Brushing frequency	Less than once daily	0.11 (0.10, 0.21)	–	–	–
	Once or more daily	reference	–	–	–
Dental visits per year	At least once	−0.04 (−0.11, 0.04)	–	–	–
	Less than once or never	reference	–	–	–
Sugar consumption	Less than once daily	−0.04 (− 0.13, 0.05)	–	–	–
	At least once daily	reference	–	–	–
Adjusted R^2^		0.084	0.087	0.092	0.090

Model 1: Oral health practices (dental visits, sugar consumption and toothbrushing) adjusted for village, age, gender and mother education

Model 2: Mothers’ parenting practices adjusted for village, age, gender and mother education

Model 3: Fathers’ parenting practices adjusted for village, age, gender and mother education

Model 4: Both mothers’ and fathers’ parenting practices adjusted for village, age, gender and mother education

The adjusted R^2^ of Models 2 to 4 that included parenting practices (for DMFT = 0.173, 0.174, 0.168, for dft = 0.403, 0.404, 0.400, for plaque index = 0.058, 0.055, 0.061 and for gingival index = 0.087, 0.092, 0.090) were similar to the adjusted R^2^ of Model 1 that included the oral health behaviors (for DMFT = 0.197, for dft = 0.421, for plaque index =0.059 and for gingival index = 0.084). Among the four oral health indicators, the adjusted R^2^ was highest for primary caries experience (Adjusted R^2^ = 0.421, 0.403, 0.404 and 0.400) and weakest for plaque index (Adjusted R^2^ = 0.059, 0.058, 0.055 and 0.061).

## Discussion

In the rural setting of the study, most children suffered from dental caries in their primary teeth, while the minority had caries in their permanent teeth. Dental plaque accumulation and gingival inflammation were moderate. There was a significant difference between the parenting practices of mothers and fathers favoring mothers in parental involvement and positive parenting and fathers in consistent disciplining, monitoring and less corporal punishment. Meanwhile, there were non-significant associations between parenting practices of both parents and oral health indicators. Mothers’ and fathers’ parenting practices explained similar amount of variation in oral health status similar to that explained by oral health behaviors. Parenting practices explained greater variation in oral health outcomes with the longest history (primary caries experience versus plaque accumulation) indicating the cumulative impact of parenting practices on children’s health. The absence of significant associations between parenting practices and oral health status indicators, however, indicate that the null hypothesis of the study cannot be rejected.

The non-significant association between parenting practices and oral health status might be related to the similarities in parenting practices across households. Parents are likely to share advice regarding parenting due to the nature of the rural setting where all families are socially connected [20]. The lack of association between parenting practices and oral health status especially plaque accumulation and gingival inflammation suggests the stronger relationship between parenting practices and chronic conditions. The earlier these conditions occur in life, and the longer time they take to develop, the stronger the association with parenting practices. On the other hand, the significant difference between parenting practices of both parents, with higher scores for mothers in positive and negative parenting may be linked to the longer time children spend with their mothers. Many children reported lower levels of fathers’ participation in daily life since fathers spend most of the day at work. The parenting practices of both parents explained equal amount of variance in oral health status indicators; both parents have equal but not similar associations with their children’s oral health.

The present findings agree with those of Aleksejūnienė and Brukienė [6] who administrated a parenting questionnaire to adolescents attending secondary schools in Lithuania and could not significantly associate parenting practices with dental plaque levels. Similarly, Ng et al. [7] could not correlate parenting styles and children’s oral health status in the United States, because the majority of parents reported the same parenting style. Dabawala et al. [10] could not confirm any significant relationship between parental-reported practices and early childhood caries in 3–5 years old children attending kindergartens in India. Alagla et al. [13] also could not find any significant association between self-reported parenting practices and dental caries in Saudi preschool children.

On the other hand, the current findings disagree with Howenstein et al. [8], who found that authoritative parenting was associated with less dental caries among 3–6 years old children who came for their first dental visit in Ohio, United States. Duijster et al. [9], similarly found that self-reported parental involvement was significantly associated with more caries-free 5–6 years old children living in the Netherlands. Also, Kumar et al. [11, 12], found that self-reported power assertion parenting was associated with higher dental caries and increased severity of gingival bleeding in 11–14 years old children in India. The disagreement between the current findings and the previous studies may be related to difference in settings where these studies were conducted in schools or pediatric dental centers in urban areas as well as to children’s age which ranged from 2 to 14 years old compared to the present study’s rural setting and age ranging from 6 to 18. Furthermore, in these previous studies, parents reported their own parenting practices which might have biased the results due to subjectivity. Our study also disagrees with De Jong-Lenters et al. [1], who recruited a sample of 5–8 years old children with their mothers, videotaped the child’s-parent interaction, and detected significant difference between children with and without dental caries in all parenting constructs except disciplining.

The strengths of the current study included using a household survey to capture details about the family environment. Interviewing the children, away from their mothers or other family members, provided less biased responses. Reporting both parents’ parenting practices allowed their direct comparison and filled a knowledge gap in oral health research by assessing differences between the impact of both parents on oral health status.

Nevertheless, there were some study limitations. It took a relatively long time to build rapport with individuals in the household. In spite of this, some households refused to participate which decreased the response rate and took more time to recruit other eligible households. In addition, interviewing younger children (aged 6–9 years) was more difficult than older ones because of their lower attention span and cognitive development constraints. This meant longer time and more training on the consistency of interviewing method among researchers. Reaching children aged 14–18 years was more difficult than younger children because most boys worked after school whereas girls were already married and moved to other households. The lower availability of these older children might have affected the age distribution among participants. High illiteracy levels were sometimes a communication barrier in some households. In addition, the present study cannot confirm a causal relationship because of its cross-sectional design. Finally, the study findings can be generalized to settings similar to rural areas in the North Western Delta of Egypt and do not apply to urban areas or areas in countries with different contextual factors.

The study findings show the association between oral health status and parenting practices which -although not statistically significant- was similar in impact to that between oral health behavior and oral health status. Future health education programs should raise parents’ awareness regarding the importance of proper parenting and its effect on children’s general and oral health. Future longitudinal studies are needed to assess the nature of the relationship between parenting practices and oral health.

## Conclusion

Parenting practices of both parents explained an amount of variation in the oral health status of children in rural areas that was similar to that explained by oral health behaviors which highlights the impact of these practices on oral health.

## Data Availability

The datasets used and/or analysed during the current study are available from the corresponding author on reasonable request.

## Abbreviations

DMFT: Decayed, missing due to caries and filled permanent teeth
dft: Decayed and filled primary teeth
APQ: Alabama Parenting Questionnaire
SD: Standard Deviation
B: Unstandardized Regression Coefficient
CI: Confidence Interval
